# Minimal important difference and patient acceptable symptom state for common outcome instruments in patients with a closed humeral shaft fracture - *analysis of the FISH randomised clinical trial data*

**DOI:** 10.1186/s12874-022-01776-6

**Published:** 2022-11-10

**Authors:** Thomas Ibounig, Joona Juurakko, Tuomas Lähdeoja, Bakir O. Sumrein, Teppo L. N. Järvinen, Mika Paavola, Clare L. Ardern, Teemu Karjalainen, Simo Taimela, Lasse Rämö

**Affiliations:** 1 Finnish Centre for Evidence-Based Orthopaedics (FICEBO), Department of Orthopaedics and Traumatology, University of Helsinki and Helsinki University Hospital, Topeliuksenkatu 5, PL266, 00029 HUS, Helsinki, Finland; 2 Finnish Centre for Evidence-Based Orthopaedics (FICEBO), Department of Hand Surgery, Central Finland Central Hospital, Keskussairaalantie 19, 40620 Jyväskylä, Finland; 3 Department of Orthopaedics and Traumatology, University of Tampere and Tampere University Hospital, Elämänaukio 2, 33520 Tampere, Finland; 4 Department of Family Practice, University of British Columbia, 5950 University Boulevard, Vancouver, V6T 1Z3 Canada

**Keywords:** Clinimetrics, Minimal important difference, MID, MCID, Patient accepted symptom state, PASS, Responsiveness, Outcome measures, DASH, Constant-Murley score, Pain, Humeral shaft fracture

## Abstract

**Background:**

Two common ways of assessing the clinical relevance of treatment outcomes are the minimal important difference (MID) and the patient acceptable symptom state (PASS). The former represents the smallest change in the given outcome that makes people feel better, while the latter is the symptom level at which patients feel well.

**Methods:**

We recruited 124 patients with a humeral shaft fracture to a randomised controlled trial comparing surgery to nonsurgical care. Outcome instruments included the Disabilities of Arm, Shoulder, and Hand (DASH) score, the Constant-Murley score, and two numerical rating scales (NRS) for pain (at rest and on activities). A reduction in DASH and pain scores, and increase in the Constant-Murley score represents improvement. We used four methods (receiver operating characteristic [ROC] curve, the mean difference of change, the mean change, and predictive modelling methods) to determine the MID, and two methods (the ROC and 75th percentile) for the PASS. As an anchor for the analyses, we assessed patients’ satisfaction regarding the injured arm using a 7-item Likert-scale.

**Results:**

The change in the anchor question was strongly correlated with the change in DASH, moderately correlated with the change of the Constant-Murley score and pain on activities, and poorly correlated with the change in pain at rest (Spearman’s rho 0.51, -0.40, 0.36, and 0.15, respectively).

Depending on the method, the MID estimates for DASH ranged from -6.7 to -11.2, pain on activities from -0.5 to -1.3, and the Constant-Murley score from 6.3 to 13.5.

The ROC method provided reliable estimates for DASH (-6.7 points, Area Under Curve [AUC] 0.77), the Constant-Murley Score (7.6 points, AUC 0.71), and pain on activities (-0.5 points, AUC 0.68).

The PASS estimates were 14 and 10 for DASH, 2.5 and 2 for pain on activities, and 68 and 74 for the Constant-Murley score with the ROC and 75th percentile methods, respectively.

**Conclusion:**

Our study provides credible estimates for the MID and PASS values of DASH, pain on activities and the Constant-Murley score, but not for pain at rest. The suggested cut-offs can be used in future studies and for assessing treatment success in patients with humeral shaft fracture.

**Trial registration:**

ClinicalTrials.gov NCT01719887, first registration 01/11/2012.

**Supplementary Information:**

The online version contains supplementary material available at 10.1186/s12874-022-01776-6.

## Background

Medical interventions should be aimed at improving patients’ health and well-being. Accordingly, patients’ symptoms and function lie at the heart of evaluating the effects of treatments. Due to their subjective nature, symptoms and function need to be assessed using patient-reported outcome measures (PROMs). Two of the most common PROMs for evaluating treatment outcome in patients with humeral shaft fractures are the Disabilities of the Arm, Shoulder, and Hand (DASH) score and Constant-Murley score [1–3]. Patients are also usually queried about the pain they experience.

But what is the minimal benefit that justifies use of a medical intervention? Over the past decades, we have witnessed increasing calls to replace statistical significance with ‘clinical relevance’ – our treatments should generate benefits that patients consider meaningful. To inform the magnitude of such effects on different outcome instruments, two important concepts have been developed: the minimal important difference (MID) [4] and the patient acceptable symptom state (PASS) [5].

The MID is “the smallest difference in score in the domain of interest which patients perceive as beneficial and which would mandate, in the absence of troublesome side effects and excessive cost, a change in the patient’s management” [4]. PASS is the symptom level above which patients consider themselves well, providing a tool for determining treatment success [5]. The main difference between MID and PASS is that the MID defines the smallest change in the given outcome that makes people feel better, and PASS defines the level at which the patient feels well.

To our knowledge, there are no previous studies reporting PASS, and only one study reporting MID estimates for two outcome measures (DASH and Constant-Murley score) in patients with humeral shaft fractures [1]. Therefore, we report the MID and PASS analyses of four outcome instruments commonly used to assess treatment outcomes after humeral shaft fractures using data from the Finnish Shaft of the Humerus (FISH) trial [3].

## Methods

### Design, setting, and participants

The FISH trial was a randomised clinical trial comparing the effectiveness of surgical treatment with open reduction and plate fixation and non-surgical treatment with functional bracing for closed humeral shaft fractures. The execution of the FISH trial has been described in detail previously [3, 6, 7]. The trial was carried out at the Helsinki and Tampere University hospitals in Finland between 2012 and 2018, and conducted in accordance with the Declaration of Helsinki. Participants provided written informed consent upon recruitment.

We included adult patients (18 years and older) with a closed, unilateral, and displaced humeral shaft fracture. Patients were excluded if they had a previous injury or a condition affecting the function of the injured upper limb, pathological fracture, other concomitant injury affecting the same upper limb, other fracture, cognitive disabilities affecting the patient compliance, or polytrauma. Characteristics of participants 6 weeks after the fracture are presented in Table 1.

**Table 1:** Participant characteristics at 6 weeks post-injury

Characteristics	Values
Total number of participants (surgery/bracing)	124 (47/77)
Completed follow-up (%)	113 (91%)
Sex, n, Female (%)	54 (44%)
Age, years, mean (SD)	47 (17)
Fracture side, n, dominant (%)	60 (48%)
Smoker, n (%)	31 (25%)
DASH score^a^, mean (SD)	46 (19)
Pain at rest^b^, mean (SD)	1.8 (1.9)
Pain on activities^b^, mean (SD)	4.9 (2.6)
Constant-Murley score^c^, mean (SD)	35 (20)

^a^The Disabilities of the Arm, Shoulder, and Hand (DASH) score from 0 to 100 (0 = best)

^b^Pain scores are 11-point Numerical Rating Scales with score from 0 to 10 (0 = best)

^c^Constant-Murley score from 0 to 100 (100 = best)

For the MID and PASS analyses, we included data from all 82 randomised participants and 42 participants who declined to be randomised (opted to choose their preferred treatment) but gave consent for prospective follow-up using the same outcomes as for the FISH trial. Accordingly, the study sample for the analyses consisted of data from 124 participants.

### Outcomes

The four outcomes analysed were the DASH score, the Constant-Murley score, and the numerical rating scale (NRS) for pain of the upper extremity, both at rest and on activities. DASH is a validated and responsive questionnaire of self-rated upper extremity disability and symptoms with a score ranging from 0 to 100 (higher is worse) [8]. The Constant-Murley score is a functional assessment score of the shoulder consisting of patients’ estimate of pain and function in daily activities, and measures of range of movement and upper extremity strength. The Constant-Murley score ranges from 0 to 100 (higher is better) [9]. The NRS for pain has been widely used to evaluate clinical pain intensity [10]. Participants are asked to rate their average pain at rest and on activities of daily living during the last 7 days on a 11-point NRS ranging from 0 to 10 (higher is worse).

As the anchor for determining both the MID and the PASS, we used the following subjective global rating question: “How satisfied are you with the overall condition of your injured upper limb and its effect on your daily life?” (for methodological details, see below). The answer options for this anchor question were from 1 to 7 in this order: “Very satisfied”, “Satisfied”, “Somewhat satisfied”, “Not satisfied nor dissatisfied”, “Somewhat dissatisfied”, “Dissatisfied”, and “Very dissatisfied”. All outcomes were collected at 6 weeks, 3, 6, 12, and 24 months after the injury.

### Data handling and analyses

#### Minimal important difference (MID)

MIDs for improvement by of each of the four outcome measures were determined using four methods.

For the three anchor-based methods, we calculated change in each outcome for each previous follow-up point by deducting the earlier score from the later score, thus a negative change in DASH and pain NRS represents improvement and conversely, a negative change in the Constant-Murley score indicates worsening.

For the *receiver operating characteristic (ROC) method*, we dichotomised the anchor question between better than the previous follow-up point (e.g., from ‘somewhat dissatisfied’ to ‘not satisfied nor dissatisfied’) and not better than the previous follow-up point. The change in the outcome score was calculated always from the previous follow-up time point to the next follow-up point (i.e., change between each follow-up). The optimal discrimination values for the outcome scores (between better and not better in subjective global rating) were determined by ROC analysis using the *closest point to top left corner method* to maximise specificity and sensitivity [11]. Nonparametric bootstrapping with 1000 replications were used to calculate the 95% confidence interval for ROC MID values [12]. To measure discrimination ability of the obtained cut-off, we calculated the area under the ROC curve (AUC) with 95% CIs by DeLong’s method by bootstrapping 2000 samples [13].

For the *mean difference of the change method,* we calculated the difference in outcomes between participants who had improved one point in the subjective global rating from those who had not improved from the previous follow-up.

For the *mean change method*, we calculated the mean change with 95% confidence intervals (CIs) for the population whose response to the anchor question (subjective global rating) was one point higher than in the previous follow-up point.

For the *predictive modelling method*, we used logistic regression analysis to calculate MIDs as described by Terluin et al. [14] In this method, a logistic regression model is used to determine an MID value that optimally predicts the probability of belonging to the improved group. We dichotomised the anchor question as better and not better as described above with the ROC method.

To assess the correlation of anchor and target outcome measures, we calculated Spearman’s rho for the change of the anchor and 1) the change in each of the outcomes, 2) prescores, and 3) postscores [15]. The 95% CIs were defined by bootstrapping 1000 samples.

#### Patient acceptable symptom state (PASS)

For PASS estimates, we used the *ROC method* and the *75th percentile method*. For the *ROC method*, we dichotomised the participants based on their responses to the subjective global rating anchor question: those responding “Very satisfied” and “Satisfied” on a 7-item Likert scale were deemed to have reached to a patient acceptable symptom state (PASS) while those responding anything between “Somewhat satisfied” to “Very dissatisfied” were deemed not to have reached the PASS. Determination of the optimal cut off and 95% CIs was carried out in the same way as for the MID.

For the *75th percentile method*, we calculated the PASS as the 25th percentile score for the Constant-Murley score, and the 75th percentile score for the DASH score and for the pain-NRS (at rest and on activities) in participants who responded either “Very satisfied” or “Satisfied” on the subjective global rating question.

#### Primary and secondary analyses

For the primary analysis, we performed the MID and PASS analyses by combining all the different time points into one analysis to obtain a sufficient number of anchor–outcome pairs. We also determined the MID values separately for every follow-up point as a secondary analysis (Tables S1 and S2 of the supplementary appendix).

## Results

In the FISH trial, 82 of 140 eligible patients were randomised to surgical (*n* = 38) or functional bracing (*n* = 44) groups. Of 58 who declined randomisation, 42 consented to follow-up (declined cohort), providing us with data from 124 participants (Table 1). Of the 42 patients in the declined cohort, nine participants chose surgery and 33 chose functional bracing. Missing data varied from 6 to 14 items at the different follow-up time points [3].

### Correlations

A change in the anchor question had good correlation with a change in the DASH score (0.51; 95% CI, 0.44 to 0.59). The change in the Constant-Murley score (-0.40; 95% CI, -0.50 to -0.31) was moderately correlated to the anchor. The correlation to pain NRS on activities (0.36; 95% CI, 0.26 to 0.47) was moderate, and poor for pain NRS at rest (0.15; 95% CI, 0.06 to 0.25). Correlations between the postscore of the outcomes and the change of the anchor ranged between -0.01 and 0.06. Correlation between the prescore of the outcomes and the change in the anchor was negative for the DASH score, pain NRS at rest, and pain NRS on activities. The correlation was positive for the Constant-Murley score (Table 2). The correlations at each time point are given in the supplementary appendix Tables S3, S4 and S5.

**Table 2:** Correlations between the change in the anchor question and outcomes

Correlation between	DASH	Pain at rest	Pain on activities	Constant-Murley score
Postscore	0.04 (-0.05 to 0.14)	0.04 (-0.06 to 0.14)	0.06 (-0.04 to 0.15)	-0.01 (-0.10 to 0.09)
Change of the outcome	0.51 (0.44 to 0.59)	0.15 (0.06 to 0.25)	0.36 (0.26 to 0.47)	-0.40 (-0.50 to -0.31)
Prescore	-0.27 (-0.36 to -0.18)	-0.09 (-0.19 to 0.01)	-0.23 (-0.32 to -0.14)	0.20 (0.11 to 0.30)

Values are Spearman’s rho with 95% CIs

*DASH* Disabilities of the Arm, Shoulder, and Hand score

### MID estimates

Depending on the method used, the MID estimates ranged from -6.7 to -11.2 for DASH, from 6.3 to 13.5 for the Constant-Murley score, and from -0.5 to -1.3 for pain-NRS on activities (Tables 3–4). Estimating MID for the pain-NRS at rest would not have been appropriate because the correlation with the anchor was too low. The MID estimates from the ROC method for DASH and the Constant-Murley score proved acceptable discrimination, while the corresponding estimates for pain-NRS on activities discriminated poorly (Table 3). The total number of anchor – outcome data pairs are shown in Table 3, and at each follow-up time point in supplementary appendix Table S4. The distribution of responses to the anchor question at different time points are shown in Fig. S1 of the supplementary appendix. The ROC curves and the MID estimates at all follow-up time points are shown in Fig. S2 and Tables S4 and S5 of the supplementary appendix.

**Table 3:** MID^a^ estimates from the ROC^b^ analyses

Outcome	MID (95% CI)	Sensitivity	Specificity	AUC^**f**^ (95% CI)	N^**g**^
DASH^c^	-6.7 (-7.9 to -5.4)	0.71	0.74	0.77 (0.73 to 0.82)	420 (172/248)
Pain on activities^d^	-0.5 (-0.5 to -0.5)	0.62	0.69	0.68 (0.63 to 0.73)	427 (178/249)
Constant-Murley score^e^	7.6 (7.4 to 13.2)	0.61	0.71	0.71 (0.66 to 0.76)	416 (172/244)

^a^Minimal important difference

^b^Receiving operating characteristics, graphs shown in Fig. S2 of the supplementary appendix

^c^The Disabilities of the Arm, Shoulder, and Hand (DASH) score from 0 to 100 (0 = optimal outcome)

^d^Pain score is 11-point Numerical Rating Scales with score from 0 to 10 (0 = optimal outcome)

^e^Constant-Murley score from 0 to 100 (100 = optimal outcome)

^f^Area under the curve

^g^N = count of anchor–outcome pairs used in the analysis (improved / not improved between consecutive time points)

**Table 4:** MID^a^ values calculated by mean difference of change, mean change, and predictive methods

Method	Mean difference of change	Mean change	Predictive^g^		
**Outcome**	**MID (95% CI)**	**MID (95% CI)**	**MID (95% CI)**	**N1**^e^	**N2**^**f**^
DASH^b^	-6.8 (-9.2 to -4.3)	-11.2 (-13.3 to -9.1)	-9.4 (-10.5 to -8.3)	105	248
Pain active^c^	-0.9 (-1.4 to -0.5)	-1.3 (-1.6 to -0.9)	-1.0 (-1.2 to -0.8)	108	249
Constant-Murley score^d^	6.3 (3.2 to 9.4)	13.5 (10.9 to 16.2)	12.1 (10.8 to 13.4)	104	244

^a^*MID* Minimal important difference. Values are MIDs with 95% CIs

^b^The Disabilities of the Arm, Shoulder, and Hand (DASH) score from 0 to 100 (0 = optimal outcome)

^c^Pain score is 11-point Numerical Rating Scales with score from 0 to 10 (0 = optimal outcome)

^d^Constant-Murley score from 0 to 100 (100 = optimal outcome)

^e^N1 = number of patients whose condition was one point better than at the previous follow-up using the 7-point Likert-scale

^f^N2 = number of patients whose condition was not better (same or worse) than at the previous follow-up using the 7-point Likert-scale

^g^Number of anchor–outcome pairs used in the predictive method are the same than with the ROC method in Table 3

### PASS estimates

PASS values showed excellent discrimination in the DASH and Constant-Murley scores in the ROC analysis. PASS values discriminated well for pain NRS on activities. It was not appropriate to define PASS value for the pain-NRS at rest due to poor correlation with the anchor. PASS values defined by the 75th percentile method were closer to the best possible score of the outcomes than the estimates obtained from the ROC method (Table 5).

**Table 5:** PASS estimates from 75th percentile method and ROC analysis

Outcome	75th percentile method	ROC method			
	PASS	PASS	Sensitivity	Specificity	AUC (95% CI)
DASH^a^	10	14	0.87	0.87	0.94 (0.92 to 0.95)
Pain on activities^b^	2.0	2.5	0.87	0.78	0.89 (0.86 to 0.91)
Constant-Murley score^c^	74	68	0.85	0.83	0.90 (0.88 to 0.93)

^a^Disabilities of the Arm, Shoulder, and Hand (DASH) score from 0 to 100 (0 = optimal outcome)

^b^Pain score is 11-point Numerical Rating Scales with score from 0 to 10 (0 = optimal outcome)

^c^Constant-Murley score from 0 to 100 (100 = optimal outcome)

*PASS* Patient acceptable symptom state

*ROC* Receiver operating characteristic

## Discussion

We calculated the MID and PASS estimates for three outcomes in adult patients with closed humeral shaft fractures. We used four methods to calculate the MID and two methods to calculate PASS.

Our MID estimates varied depending on the method used. The change in DASH score had a good correlation, and the change of Constant-Murley score and pain on activities had moderate correlations with the change in anchor question. Pain at rest did not correlate with the anchor question and therefore we were not able to estimate MID or PASS for pain at rest. Taken together, these results indicated credible MID estimates. The ROC method for cut-off values of the MID of both DASH (﻿-6.7﻿ points) and Constant-Murley (7.6 points) scores had an acceptable discrimination. Pain on activities (-0.5 points) discriminated poorly with the ROC method.

The PASS values with the ROC method for DASH (14 points) and Constant-Murley score (68 points) had excellent discrimination. The discrimination was good with the pain on activities (2.5 points). The 75th percentile method yielded more stringent limits for PASS in all the outcomes (DASH, 10 points; the Constant-Murley score, 74 points; pain on activities, 2 points).

We suggest that differences smaller than the smallest point estimates of the MIDs from this study are unlikely to be clinically meaningful. Conversely, differences above the upper limits are very likely to be clinically important to patients. Depending on the potential benefits and inherent risks of treatment methods, researchers may choose either the lower or upper limit of the suggested MID when interpreting the clinical relevance of treatment effects. For PASS, the upper point estimate depicts the cut-off above which the patients are very likely to be satisfied with the treatment outcome and conversely, the lower point estimate reflects the level below which the patients are unlikely to be satisfied.

We identified one previous prospective comparative study on the MID of two different outcomes in patients with humeral shaft fractures reporting the MID of 6.7 points for DASH and 6.1 points for the Constant-Murley score [1]. We could not identify a previous study reporting PASS estimates for patients with humeral shaft fractures. Our estimate for the MID for pain on activities is smaller than in degenerative shoulder conditions [16, 17]. However, due to moderate correlation in pain on activities, our result should be interpreted with caution.

We decided to use a prospective anchor question for our analyses (i.e., patients reported their current symptom state using the subjective global rating as opposed to comparing it to baseline status), which is the method used often in the MID analyses for degenerative conditions. In a trauma setting, it is not possible to obtain reliable baseline data prior the injury. Our approach may be less susceptible to recall bias as the participants did not have to remember their symptoms state several months ago—a task that people tend to fail in [18, 19].

A strength of our study is high internal validity as we used prospective homogenous data from a randomised clinical trial performed by experienced research personnel with little missing data. We also used the most common outcome instruments to assess the outcome of treatment in patients with upper extremity injuries and the methods for obtaining several MID and PASS estimates. In addition, our determination to analyse the MID and PASS was published in the protocol article, prior to any access to trial data [7].

### Limitations

An obvious limitation of our study is that the results are obtained from a randomised clinical trial with stringent inclusion and exclusion criteria (i.e., adult patients with closed, unilateral humeral shaft fracture without severe comorbidities or compliance problems). Thus, our results may not be directly applicable to all patients with this injury. Second, the ROC analyses can be biased if the proportion of improved participants is markedly different from 50% [20]. However, in our study there were about 420 follow-up intervals and in approximately 250 intervals the patients did not experience improvement, making a marked bias in the estimates unlikely.

### Future directions

Both the MID and PASS are valuable tools both in medical research and clinical practice. The MID provides a tool for future trial sample size calculations. However, when contemplating different treatment methods during shared decision-making in clinical settings, the concept of PASS may be more understandable for patients [21]. The clinician might consider informing the patient about the probable proportion of patients reaching PASS (i.e., feeling well, with an experience of successful treatment) with different treatment options.

## Conclusions

We provide credible estimates for the MID and PASS for adult patients with humeral shaft fractures including several of the most used methods and outcomes. Depending on the application, the upper or lower limit of the established MIDs and PASS values should be chosen. The MID might be more useful especially for scientific purposes (i.e., sample size calculation), whereas the PASS concept is—in addition to scientific applications—more understandable to patients, and accordingly, we advocate its use as a more appropriate measure for gauging treatment success in patients with a humeral shaft fracture.

## Supplementary Information

**Additional file 1.**

## Data Availability

FISH trial data are not publicly available owing to data privacy issues, but access to the anonymised dataset can be obtained from the corresponding author on reasonable request.

## Abbreviations

AUC: Area under curve
CI: Confidence interval
DASH: the Disabilities of arm, Shoulder, and Hand score
FISH: Finnish Shaft of the Humerus trial
MID: Minimal important difference
NRS: Numerical rating scale
PASS: Patient acceptable symptom state
PROM: Patient-reported outcome measure
ROC: Receiver operating characteristic
