# Supplementary material for: Minimal important difference and patient acceptable symptom state for common outcome instruments in patients with a closed humeral shaft fracture - analysis of the FISH randomised clinical trial data
Source: BMC Med Res Methodol. 2022 Nov 10;22:291. doi: 10.1186/s12874-022-01776-6 (PMC9650807; doi:10.1186/s12874-022-01776-6)
Supplement: Supplementary file 1 — Additional file 1. [file 12874_2022_1776_MOESM1_ESM.docx]

**SUPPLEMENT**

**Minimal important difference and patient acceptable symptom state for common outcome instruments in patients with a closed humeral shaft fracture**

***Analysis of the FISH randomised clinical trial data***

Thomas Ibounig, Joona Juurakko, Tuomas Lähdeoja, Bakir O. Sumrein, Teppo LN Järvinen, Mika Paavola, Clare L. Ardern, Teemu Karjalainen, Simo Taimela, Lasse Rämö

[Fig. S1. Responses to anchor question: “How satisfied are you with the overall condition of your injured upper limb and its effect on your daily life?" 2](#_Toc114949212)

[Fig. S2. ROC curves for the outcomes using data from all follow-up time points 3](#_Toc114949213)

[Table S1. MID estimates of all follow-up time points from the ROC analysis 4](#_Toc114949214)

[Table S2. MID values of all follow-up time points using mean change, mean difference of change, and predictive methods 5](#_Toc114949215)

[Table S3. Correlations between the change in the anchor question and outcomes postscores 6](#_Toc114949216)

[Table S4. Correlations between the change in the anchor question and the change in the outcome 6](#_Toc114949217)

[Table S5. Correlations between the change in the anchor question and outcomes prescores 6](#_Toc114949218)

Fig. S1. Responses to anchor question: “How satisfied are you with the overall condition of your injured upper limb and its effect on your daily life?"

| ***1*** | ***Very satisfied*** |
| --- | --- |
| ***2*** | ***Satisfied*** |
| ***3*** | ***Somewhat satisfied*** |
| ***4*** | ***Not satisfied or dissatisfied*** |
| ***5*** | ***Somewhat dissatisfied*** |
| ***6*** | ***Dissatisfied*** |
| ***7*** | ***Very dissatisfied*** |

*
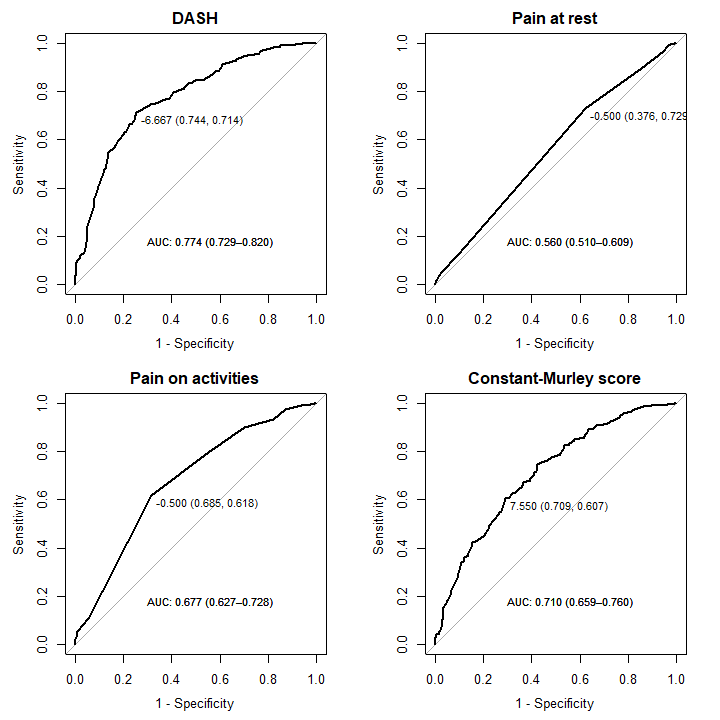
*Fig. S2. ROC curves for the outcomes using data from all follow-up time points

Table S1. MID estimates of all follow-up time points from the ROC analysis

| **Outcome measure** | **MID (95% CI)** | **Sensitivity** | **Specificity** | **AUC (95% CI)** | **N*** |
| --- | --- | --- | --- | --- | --- |
| **12 weeks** |  |  |  |  |  |
| DASH | -17.3 (-19.6 to -12.1) | 0.72 | 0.69 | 0.74 (0.65 to 0.84) | 107 (54/53) |
| Pain active | -1.5 (-1.5 to 0.5) | 0.67 | 0.56 | 0.63 (0.53 to 0.74) | 111 (57/54) |
| Constant Score | 16.9 (13.1 to 21.5) | 0.60 | 0.69 | 0.71 (0.61 to 0.80) | 110 (55/55) |
| **26 weeks** |  |  |  |  |  |
| DASH | -7.9 (-12.9 to -7.9) | 0.59 | 0.82 | 0.73 (0.63 to 0.82) | 104 (55/49) |
| Pain active | -0.5 (-1.5 to -0.5) | 0.51 | 0.74 | 0.71 (0.61 to 0.81) | 106 (57/49) |
| Constant Score | 18.8 (3.4 to 18.8) | 0.8 | 0.45 | 0.65 (0.54 to 0.75) | 105 (56/49) |
| **52 weeks** |  |  |  |  |  |
| DASH | -4.2 (-6.7 to -2.9) | 0.73 | 0.76 | 0.82 (0.73 to 0.91) | 104 (37/67) |
| Pain active | -0.5 (-0.5 to -0.5) | 0.70 | 0.74 | 0.71 (0.61 to 0.81) | 105 (38/67) |
| Constant Score | 7.6 (5.6 to 12.5) | 0.67 | 0.62 | 0.67 (0.56 to 0.79) | 101 (37/64) |
| **104 weeks** |  |  |  |  |  |
| DASH | -2.9 (-4.2 to -0.8) | 0.81 | 0.58 | 0.69 (0.56 to 0.82) | 105 (26/79) |
| Pain active | -0.5 (-0.5 to -0.5) | 0.72 | 0.46 | 0.62 (0.50 to 0.74) | 105 (26/79) |
| Constant Score | 3.9 (1.9 to 9.3) | 0.61 | 0.67 | 0.64 (0.50 to 0.77) | 100 (24/76) |
| **All timepoints** |  |  |  |  |  |
| DASH | -6.7 (-7.9 to -5.4) | 0.71 | 0.74 | 0.77 (0.73 to 0.82) | 420 (172/248) |
| Pain active | -0.5 (-0.5 to -0.5) | 0.62 | 0.69 | 0.68 (0.63 to 0.73) | 427 (178/249) |
| Constant Score | 7.6 (7.4 to 13.2) | 0.61 | 0.71 | 0.71 (0.66 to 0.76) | 416 (172/244) |
|  |  |  |  |  |  |

*DASH score from 0 to 100 (0 = optimal outcome)*

*Pain scores are NRS with score from 0 to 10 (0 = optimal outcome)*

*Constant-Murley Score from 0 to 100 (100 = optimal outcome)*

** N = count of anchor–outcome pairs used in the analysis (improved / not improved between the current and previous time points)*

Table S2. MID values of all follow-up time points using mean change, mean difference of change, and predictive methods

| **Outcome measure** | **Mean difference of change** | **Mean change** | **Predictive** | **N1** | **N2** |
| --- | --- | --- | --- | --- | --- |
|  |  |  |  |  |  |
| **12 weeks** |  |  |  |  |  |
| DASH | -8.4 (-14.4 to -2.3) | -19.5 (-24.5 to -14.7) | -16.5 (-18.7 to -14.2) | 28 | 53 |
| Pain active | -1.1 (-2.3 to 0.0) | -1.7 (-2.5 to -0.9) | -1.2 (-1.7 to -0.7) | 30 | 54 |
| Constant Score | 11.6 (4.5 to 18.8) | 24.9 (19.7 to 30.2) | 19.6 (16.6 to 22.6) | 28 | 55 |
|  |  |  |  |  |  |
| **26 weeks** |  |  |  |  |  |
| DASH | -3.8 (-8.2 to 0.7) | -11.2 (-14.2 to -8.3) | -12.0 (-14.2 to -9.9) | 28 | 49 |
| Pain active | -0.5 (-1.5 to 0.5) | -1.3 (-2.1 to -0.6) | -1.4 (-1.8 to -1.0) | 29 | 49 |
| Constant Score | 0.0 (-5.4 to 5.4) | 9.8 (6.3 to 13.4) | 13.8 (11.1 to 16.6) | 28 | 49 |
|  |  |  |  |  |  |
| **52 weeks** |  |  |  |  |  |
| DASH | -6.6 (-9.5 to -3.7) | -8.0 (-10.3 to -5.8) | -5.3 (-6.2 to -4.2) | 27 | 67 |
| Pain active | -1.0 (-1.7 to -0.4) | -1.2 (-1.7 to -0.8) | -0.8 (-1.1 to -0.6) | 27 | 67 |
| Constant Score | 5.6 (0.2 to 10.9) | 10.7 (6.1 to 15.3) | 8.6 (6.5 to 10.7) | 27 | 64 |
|  |  |  |  |  |  |
| **104 weeks** |  |  |  |  |  |
| DASH | -3.9 (-8.1 to 0.2) | -4.3 (-8.0 to -0.6) | -2.7 (-4.2 to -1.2) | 22 | 79 |
| Pain active | -0.7 (-1.4 to 0.1) | -0.7 (-1.3 to -0.1) | -0.4 (-0.7 to -0.1) | 22 | 79 |
| Constant Score | 4.0 (-0.4 to 8.5) | 6.8 (2.9 to 10.7) | 4.8 (2.9 to 6.6) | 21 | 76 |
|  |  |  |  |  |  |
| **All timepoints** |  |  |  |  |  |
| DASH | -6.8 (-9.2 to -4.3) | -11.2 (-13.3 to -9.0) | -9.4 (-10.5 to -8.3) | 105 | 248 |
| Pain active | -0.9 (-1.4 to -0.5) | -1.3 (-1.6 to -0.9) | -1.0 (-1.2 to -0.8) | 108 | 249 |
| Constant Score | 6.3 (3.2 to 9.4) | 13.5 (10.9 to 16.2) | 12.1 (10.8 to 13.4) | 104 | 244 |
|  |  |  |  |  |  |

*Values are MIDs with 95 % CI.*

*DASH score from 0 to 100 (0 = optimal outcome)*

*Pain scores are NRS with score from 0 to 10 (0 = optimal outcome)*

*Constant-Murley Score from 0 to 100 (100 = optimal outcome)*

*N1 = patients whose condition was one point better on the 7-item Likert scale compared to previous follow-up visit*

*N2 = whose condition was not better compared to previous follow-up visit*

Table S3. Correlations between the change in the anchor question and outcomes postscores

| **Post score at** | **Dash score** | **Pain rest** | **Pain active** | **Constant Score** |
| --- | --- | --- | --- | --- |
| 12 weeks | 0.22 (0.051 to 0.4) | 0.19 (0.025 to 0.38) | 0.30 (0.14 to 0.49) | -0.30 (-0.48 to -0.14) |
| 26 weeks | 0.18 (0.00 to 0.39) | -0.05 (-0.25 to 0.15) | 0.16 (-0.04 to 0.35) | -0.15 (-0.35 to 0.03) |
| 52 weeks | 0.12 (-0.06 to 0.32) | 0.00 (-0.21 to 0.2) | 0.05 (-0.14 to 0.25) | 0.04 (-0.12 to 0.23) |
| 104 weeks | 0.15 (-0.03 to 0.35) | 0.23 (0.03 to 0.45) | 0.11 (-0.09 to 0.31) | -0.22 (-0.42 to -0.03) |
| All time points | 0.04 (-0.05 to 0.14) | 0.04 (-0.06 to 0.14) | 0.06 (-0.04 to 0.15) | -0.01 (-0.10 to 0.09) |

*Values are Spearman’s rho with 95 % CI.*

Table S4. Correlations between the change in the anchor question and the change in the outcome

| **Change from** | **Dash score** | **Pain rest** | **Pain active** | **Constant Score** |
| --- | --- | --- | --- | --- |
| 6 to 12 weeks | 0.50 (0.37 to 0.66) | 0.08 (-0.11 to 0.28) | 0.25 (0.09 to 0.44) | -0.4 (-0.58 to -0.24) |
| 12 to 26 weeks | 0.49 (0.34 to 0.68) | 0.19 (0.00 to 0.40) | 0.32 (0.12 to 0.51) | -0.38 (-0.58 to -0.22) |
| 26 to 52 weeks | 0.52 (0.37 to 0.72) | 0.13 (-0.08 to 0.34) | 0.47 (0.31 to 0.67) | -0.31 (-0.52 to -0.13) |
| 52 to 104 weeks | 0.29 (0.11 to 0.52) | 0.15 (-0.06 to 0.38) | 0.29 (0.11 to 0.50) | -0.25 (-0.49 to -0.05) |
| All time points | 0.51 (0.44 to 0.59) | 0.15 (0.06 to 0.25) | 0.36 (0.26 to 0.47) | -0.40 (-0.50 to -0.31) |

*Values are Spearman’s rho with 95 % CI.*

Table S5. Correlations between the change in the anchor question and outcomes prescores

| **Pre score at** | **Dash score** | **Pain rest** | **Pain active** | **Constant Score** |
| --- | --- | --- | --- | --- |
| 6 weeks | -0.14 (-0.33 to 0.03) | 0.15 (-0.05 to 0.35) | 0.02 (-0.17 to 0.20) | 0.05 (-0.16 to 0.25) |
| 12 weeks | -0.19 (-0.39 to 0.02) | -0.19 (-0.40 to -0.01) | -0.21 (-0.40 to -0.04) | 0.12 (-0.10 to 0.32) |
| 26 weeks | -0.27 (-0.47 to -0.01) | -0.14 (-0.35 to 0.07) | -0.32 (-0.51 to -0.16) | 0.20 (0.02 to 0.41) |
| 52 weeks | -0.09 (-0.29 to 0.09) | 0.05 (-0.17 to 0.26) | -0.05 (-0.23 to 0.14) | -0.09 (-0.28 to 0.13) |
| All time points | -0.27 (-0.36 to -0.18) | -0.09 (-0.19 to 0.01) | -0.23 (-0.32 to -0.14) | 0.20 (0.11 to 0.30) |

*Values are Spearman’s rho with 95 % CI.*
